# Static postural balance study in patients with vestibular disorders using a three dimensional eletromagnetic sensor system

**DOI:** 10.1590/S1808-86942012000300002

**Published:** 2015-10-14

**Authors:** David Greco Varela, Jose Ailton Oliveira Carneiro, José Fernando Colafêmina

**Affiliations:** a MSc (PhD student at the Department of Ophthalmology, Otorhinolaryngology and Head and Neck Surgery of the FMRP-USP, Professor of Otorhinolaryngology at the FTC school).; b MSc (PhD student at the Department of General Practi ce Medicine of the Medical School of Ribeirão Preto - University of São Paulo - FMRP-USP, Ribeirão Preto/SP, Brazil).; c PhD; Professor at the Department of Ophthalmology, Otorhinolaryngology and Head and Neck Surgery of the FMRP-USP. Department of Otorhinolaryngology, Ophthalmology and Head and Neck Surgery – Medical School of Ribeirão Preto.

**Keywords:** dizziness, labyrinth diseases, posture, vertigo

## Abstract

The vestibular-ocular reflex assessment is important, but not enough. Tridimensional electromagnetic sensor systems represent a new method to assess posturography.

**Aim:**

To assess body sway in healthy subjects who had positive Dix Hallpike and Epley maneuvers and with other vestibular dysfunctions by means of a three-dimensional system. Study design: Prospective.

**Materials and Methods:**

We had 23 healthy women, 15 with peripheral vestibular dysfunction found upon caloric test and 10 with positive Epley and Dix Hallpike maneuvers. All tests performed in the following positions: open and closed eyes on stable and unstable surfaces.

**Results:**

With the Eyes Open and on a stable surface, *p* < 0.01 between the control group and the one with peripheral vestibular dysfunction in all variables, except the a-*p* maximum, full speed and mediolateral trajectory velocity, which had a *p* < 0.01 between the group with vestibular dysfunction and controls in all positions. The group with positive Epley and Dix Hallpike maneuvers had *p* < 0.01 at full speed and in its components in the x and y in positions with open and eyes closed on an unstable surface.

**Conclusion:**

The tridimensional electromagnetic sensors system was able to generate reliable information about body sway in the study volunteers.

## INTRODUCTION

The early detection of postural dysfunctions is paramount to promote the proper interventions to patients with balance disorders. The proprioceptive component added to the information from other sensorial system enriches clinical reasoning and leads to a better work from the healthcare team^1^, ^2^.

In labyrinthine disorders, it is very important to assess the oculovestibular reflex (OVR); however, it is not enough to reach a precise diagnosis^3^. The vestibulospinal reflex (VSR) analysis and integration with the visual and proprioceptive information make the test more complete^4^, ^5^. The Balance and Social Integration Clinical Test (BSICT), employed in the computerized dynamics posturography, was developed with the aim of identifying the contribution of the three main sensorial systems associated with balance (visual, vestibular and somatosensory)^6^, ^7^, ^8^. This test tries to isolate the many sensorial contributions by means of surface and vision distortion or removal^6^, ^9^, ^10^. Nonetheless, one of the main difficulties concerning the use of computerized posturography is the cost of the equipment (force platform) and the restrictions concerning its transportation^1^.

Among the recent Technologies which study body sway, the tridimensional electromagnetic sensor system^1^, ^11^ has, as potential advantage in relation to the force platform, the lower cost and its better portability. Posturography studies have already been carried out in geriatrics, orthopedics, rheumatology, neurology and endocrinology^12^, ^13^. The employment of this method in otorhinolaryngology may add to the information from the tests routinely employed in this medical specialty.

The present study did not have any financial support or sponsors, and it is the first investigating individuals with labyrinthine disorders using a system of tridimensional electromagnetic sensors. Considering the lack of proper tools able to quantify body sway in a more objective way, it is valid to employ a new approach in healthy persons, in volunteers with Epley and Dix-Hallpike maneuvers and in volunteers with other peripheral labyrinth disorders detected by electronystagmography and the caloric test.

## MATERIALS AND METHODS

This is a cross-sectional cohort study.

The group of cases was recruited from a reference otorhinolaryngology ward from a medical school, between October, 2010 and October of 2011. From a total of 1,344 adults seen, 69 women between 18 and 59 years complained of dizziness; they denied using prior medication for the treatment of this health problem and had some acute symptoms. Of these patients, five gave up or were unable to finish the tests proposed by the study. Of the remaining 64 patients, 15 had caloric tests and electronystagmography matching peripheral vestibular dysfunction and 10 had positive Epley's and Dix-Hallpike maneuvers; and electronystagmography tests within normal ranges.

The group 1 of cases was made up of 15 volunteer women with electronystagmography and caloric tests compatible with vestibular dysfunction and without changes upon the Epley and Dix-Hallpike maneuvers.

Considering that the posterior semicircular canal BPPV is the most common cause of labyrinth disease^4^, that its diagnosis is based on performing specific maneuvers and knowing that electronystagmography has a low sensitivity concerning its detection, we chose to make group 2 of cases with those volunteers who would have a clinical history of rotational dizziness lasting for seconds, with positive Dix-Hallpike and Epley maneuvers and with electronystagmography test within normal ranges.

The components of group 2 cases were only known after having positive Dix-Hallpike and Epley maneuvers, which were carried out in all participants after the postural sway and electronystagmography tests.

The control group was made up of 23 volunteers who participated in health-promoting talks and activities led by the Medical School in the years of 2010 and 2011. They were between 18 and 59 years of age, in good health, should not have a prior diagnosis of balance disorder, labyrinthine, visual, orthopedic, neurologic and psychiatric disorders.

We took off the study those patients with chronic or acute otitis media seen upon otoscopy, intense and uncorrected visual loss evaluated by means of the Snellen test, bone or muscle diseases which did not enable the examinee to remain standing during the tests or who did not finish any stage of the study. Other reasons for exclusion were: the report of central neurological diseases (epilepsies, a past of strokes, demyelinating diseases, and others), symptomatic peripheral diseases, psychiatric disorders under treatment with medication and symptomatic bone and muscle disorders. All volunteers were educated on the study and signed the consent form in the protocol #2082/2010, approved by the Ethics in Research Committee of the institution where the study was carried out.

We measured the weight and height of the volunteers in order to calculate the body mass index (BMI). We used a digital scale (from Filizola) with a variation of 0.1 kg, with the individual dressing light clothes and barefoot. The height was measured with the vertical stadiometer, graduated every 0.5 cm.

All participants in the study were instructed to refrain from having coffee, chocolate, black tea, mate tea, sodas, smoking, alcoholic drinks, sedatives, antiallergic agents, tranquillizers and analgesic medication for two days before the tests. Other medications of continuous use were not interrupted. We carried out the following exams: otoscopy, oroscopy, rhinoscopy, TMJ assessment, neck test and clinical labyrinthine tests (Romberg, Untenberg, Dix-Hallpike, Epley; motor coordination tests and proprioceptive sensitivity). We also did: tonal and vocal audiometry, visual assessment with and without correction using the Snellen chart.

**Figure 1:**
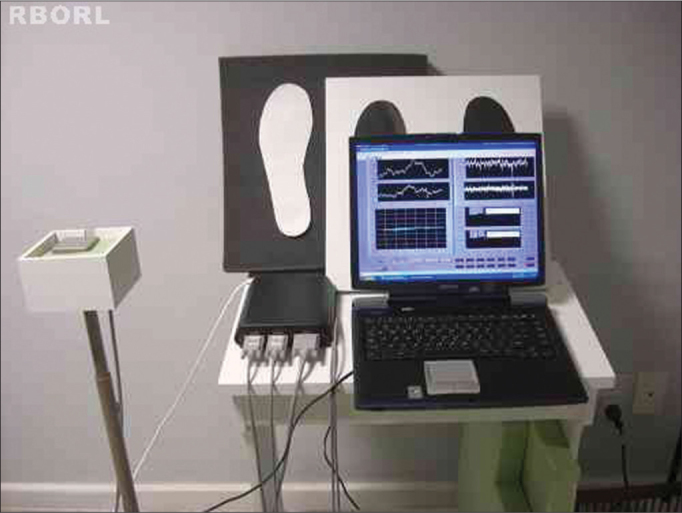
Equipment utilized in the study: Polhemus, notebook and test platforms.

**Figure 2:**
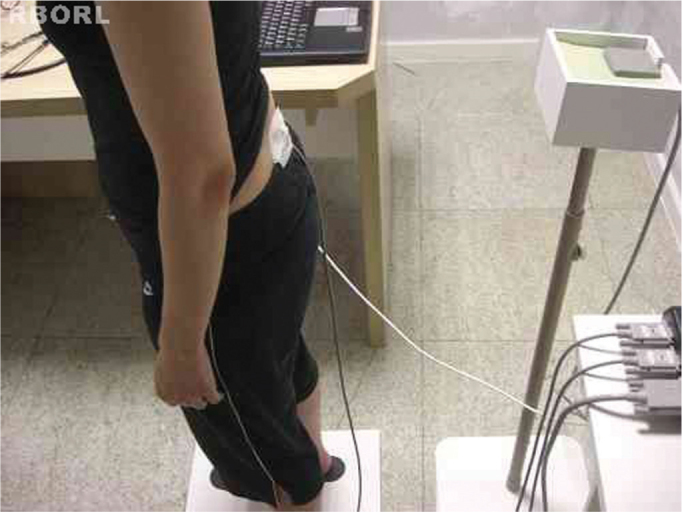
Sensor fixed between S1 and S2.

Figure 1 shows the tridimensional Polhemus^®^ Patriot (Polhemus, USA) electromagnetic sensors. Such system is made up of three perpendicular coils (22.9 mm x 28.3 mm x 15.2 mm) connected to an amplifier and it is based on the emission and detection of a magnetic field, with a 2 mm accuracy (absolute) and approximately 0.1 mm (relative). Figure 2 depicts the fixation of the receptor sensor on the sacral region (mass center location). The transmitter sensor was placed on a support detached from the body at a distance of approximately 40 cm at the same height of the sensor receptor^1^. The data acquired by the system was transferred to a notebook computer at a rate of 60 samplings per second, by means of a USB connection, having a control interface and processing developed in the LabView 8.0 environment.

We considered the maximum anteroposterior shift (cm), the greater movement amplitude in the anteroposterior direction (a-*p*) and in the maximum medio-lateral (m-l) direction. The total trajectory was defined as total body shift (cm) during the data acquisition time in the x, y and z axes. The total velocity (cm/s) was defined as the total trajectory in the three dimensions divided by time. The velocities in the anteroposterior (a-*p*) and mediolateral (m-l) directions were calculated according to the trajectory in the different axes in relation to time (cm/s).

During the assessment of body sway, the volunteers remained standing up with their arms hanging along the body and their feet slightly apart on a reference surface. Moreover, they were instructed to keep still, not moving their upper limbs, heels and feet on the wooden platform (stable) of 1 cm in height, 50 cm long and 50 cm wide and, later, on a foam surface (unstable), with a density of 28kg/m^3^, 5 cm height, 50 cm long and 50 cm wide. The test was carried out in four sensorial situations during 90 seconds for each position, in the following order: 1) open eyes and stable surface (OESS); 2) closed eyes and stable surface (CESS), 3) open eyes and unstable surface (OEUS) and 4) closed eyes and unstable surface (CEUS). With the eyes open, all the individuals were instructed to keep their eyes fixed at a point placed at 1.5 m in front of them.

Electronystagmography and caloric testing were done after posturography. We used the CONTRONIC - SCE Nistamus – electronystagmography; 5.11 computerized vector-electronystagmography and E96 otocalorimeter otoneurological water-stimulator. We investigated: a) spontaneous nystagmus, b) semi-spontaneous nystagmus, c) saccadic movement study, d) pendulum tracking study, e) optokinetic signs, f) pre and post-caloric nystagmus. We processed the responses given by the slow component angular velocity (SCAV) in each test. In the caloric test we also calculated labyrinth predominance (LP) and directional preponderance (DP). At the end, the test was characterized as normal, changed in the periphery and changed centrally. We took off the group 1 of cases those patients with tests suggesting central nervous system changes, changes in eye movement and those who were unable to finish the test.

All volunteers complaining of dizziness received the result from their tests and continued being followed in the Otolaryngology reference ward.

We carried out a descriptive statistical analysis, calculating the mean and the standard deviation. The Kolmogorov-Smirnov test was used in order to test data normality. In order to compare the intergroup body sway variables, we used the Kruskall Wallis test. We also employed the Dunn test for multiple comparisons between the groups, and we used the significance level of 5%. The data was processed electronically, using the Statistical Package for the Social Science^®^ (SPSS) version 16.0 software.

## RESULTS

Table 1 depicts the physical characteristics of the investigated samples. Table 2 shows the intergroup body sway variables in the different sensorial situations. In the OESS situation, we noticed a significant difference between the control group and the group with vestibular dysfunction in all the variables (*p* < 0.01), except for the maximum a-*p* shift (*p* = 0.15).

**Table 1:** Physical characteristics of the samples studied.

	Healthy volunteers (n=23)	Other peripheral vestibular disorders (n=15)	Positive Dix-Hallpike and Epley maneuvers (n=10)	p-value
Age (years)	33.0±10.1	45.3±11.3	48.6±11.3	<0.001
Weight (kg)	65.0±14.3	68.0±12.0	65.8±10.0	0.78
Height (cm)	1.61±0.06	1.57±0.08	1.59±0.06	0.41
BMI^a^ (kg/m^2^)	25.1±5.3	27.4±5.0	26.0±5.0	0.38

a BMI Body Mass Index.

**Table 2:** Values of the stabilometric variables of the three groups in different sensorial conditions.

Variables	Sensorial situation	Healthy volunteers (n=23)	Other peripheral vestibular disorders (n=15)	Positive Dix-Hallpike and Epley maneuvers (n = 10)	p-value
Shift	OESS^d^	1.33 ± 0.4	1.67 ± 0.7	1.11 ± 0.6	0.15
Maximum	CESS^e^	1.35 ± 0.43	1.65 ± 0.65	1.64 ± 0.75	0.16
anteroposterior	OEUS^f^	1.44 ± 0.44	1.78 ± 0.63	1.7 ± 0.8	0.08
(cm)	CEUS^g^	1.6 ± 0.57	2.1 ± 0.6	2.0 ± 0.76	0.07

Shift	OESS^d^	0.62 ± 0.4	1.17 ± 0.9	0.84 ± 0.4	0.02^a^
Maximum	CESS^e^	0.70 ± 0.2	1.10 ± 1.0	0.90 ± 0.4	0.42
Mid-lateral	OEUS^f^	1.01 ± 0.42	1.30 ± 0.77	1.26 ± 0.47	0.22
(cm)	CEUS^g^	1.04 ± 0.4	1.4 ± 0.8	1.4 ± 0.8	0.24

Trajectory	OESS^d^	23.1 ± 9.8	45.5 ± 36.5	31.8 ± 13.1	0.01^a^
anteroposterior	CESS^e^	28.5 ± 11.2	49.7 ± 37.4	38.5 ± 18.5	0.06
(cm)	OEUS^f^	24.5 ± 6.5	42.7 ± 30.0	38.9 ± 18.6	< 0.01^a^
	CEUS^g^	29.6 ± 8.3	53.1 ± 32.7	44.1 ± 17.3	< 0.01^a^

Trajectory	OESS^d^	20.0 ± 5.6	47.0 ± 30.5	36.1±18	< 0.01^a^
Mid-lateral	CESS^e^	23.9 ± 8.4	51.9 ± 33.4	39.9 ± 20.5	< 0.01^a^
(cm)	OEUS^f^	23.5 ± 6.0	45.1 ± 21.4	42.4 ± 18.1	< 0.01^a^, ^b^
	CEUS^g^	26.4 ± 7.3	52.1 ± 25.7	43.4 ± 16.4	< 0.01^a^, ^c^

Velocity	OESS^d^	0.25 ± 0.1	0.50 ± 0.4	0.35 ± 0.26	< 0.01^a^
anteroposterior	CESS^e^	0.26 ± 0.1	0.55 ± 0.41	0.42 ± 0.20	0.06
(cm)	OEUS^f^	0.27 ± 0.07	0.47 ± 0.33	0.43 ± 0.20	< 0.01^a^
	CEUS^g^	0.33 ± 0.1	0.59 ± 0.36	0.49 ± 0.25	< 0.01^a^

Velocity	OESS^d^	0.22 ± 0.6	0.52 ± 0.3	0.40 ± 0.20	< 0.01^a^
Mid-lateral	CESS^e^	0.26 ± 0.1	0.58 ± 0.37	0.44 ± 0.22	< 0.01^a^
(cm)	OEUS^f^	0.26 ± 0.06	0.50 ± 0.23	0.47 ± 0.21	< 0.01^a^, ^b^
	CEUS^g^	0.29 ± 0.01	0.58 ± 0.28	0.48 ± 0.18	< 0.01^a^, ^b^

Velocity	OESS^d^	0.46 ± 0.16	1.03 ± 0.60	0.76 ± 0.31	< 0.01^a^
Total	CESS^e^	0.55 ± 0.20	1.13 ± 0.65	0.0 ± 0.42	< 0.01^a^
(cm/s)	OEUS^f^	0.50 ± 0.12	1.0 ± 0.5	0.9 ± 0.41	< 0.01^a^, ^b^
	CEUS^g^	0.57 ± 0.15	1.13± 0.52	0.95 ± 0.36	< 0.01^a^, ^b^

a difference between groups A and B (*p* < 0.05);

b difference between groups A and C (*p* < 0.05);

c difference between groups B and C (*p* < 0.05);

d OESS: open eyes, stable surface;

e CESS: closed eyes, stable surface;

f OEUS: open eyes, unstable surface;

g CEUS: closed eyes, unstable surface.

In the CESS situation, when the visual information was withdrawn, significant differences were seen between the control group and the group with vestibular dysfunction in all the variables (*p* < 0.01), except in the maximum a-p shift (*p* = 0.16) and maximum m-l shift (*p* = 0.42).

In the OEUS and CEUS situations, when the support surface was unstable and there was a reduction of the proprioceptive information, we noticed a significant difference between the control group and group 1 with vestibular dysfunction in all the variables (*p* < 0.01), except for variables maximum a-*p* shift (*p* = 0.08 and 0.07) and maximum m-l shift (*p* = 0.22 and 0.24). In these same situations, we also noticed a significant difference between the control group and group 2 with BPPV in the m-l trajectory and total; and m-l velocity and total (*p* < 0.01).

## DISCUSSION

The present study assessed body sway in adults in the age range between 18 and 59 years. It is known that 50% of the vestibular disorders are diagnosed in people older than 60 years^4^; however, there is the risk of underdiagnoses in all age ranges, especially when the investigation of the proprioceptive and visual systems is partial.

Analyzing the cases and controls did not show differences as to height and weight; however, there was a difference as to age composition. Nevertheless, the case and control groups were classified as middle-aged adults. Upon comparing youngsters and elderly, it is known that the former have better stability because of a better control of body segments (ankle, hip and trunk); nonetheless, the sample analyzed did not make such comparison. Freitas^14^, in his study on postural control, discusses human chronological characteristics.

The pressure center variation is the most utilized parameter by the force platforms for static and dynamics posturographic analysis^15^. Tridimensional electromagnetic sensor systems, such as the Polhemus, utilize the direct variation of the mass center in order to calculate sway velocity and maximum shift in the anteroposterior and medio-lateral directions^12^, ^16^. In the present study, because of positioning the sensor always in the region near the mass center of each participant, it was not necessary to normalize the postural sway variables in relation to height.

According to Raymarkes et al.^15^, body sway is the best isolated parameter to prevent falls. In the current study, the data captured by the static posturography indicates greater changes in Group 1, with other peripheral vestibular dysfunctions than in Group 2. Such finding was expected, because the description of the fits in patients with changed Epley and Dix-Hallpike maneuvers are milder and shorter^17^.

The increased body sway velocity in groups 1 and 2 of cases in situations OEUS and CEUS (total and medio-lateral velocities) may indicate a clear situation of unbalance. Studies carried out with obese elderly women and patients with osteoporosis were in agreement as far as the analysis of this parameter is concerned^16^, ^18^. Conversely, the study carried out using the Polhemus^®^ in seated elderly, showed a reduction in this velocity^13^, it may be because of a different mechanism of compensation, in which the ankle and hip had a lower participation and the vibrating body segment analyzed was smaller.

According to revision studies, static posturography, still requires standardization for measurements and thus reproducibility^15^, ^19^. Many authors agree with the findings of the current paper, as to the statement that when one increases the sway velocity in sick individuals, there is a correlation with the risk of falls and unbalance^16^, ^20^.

Dynamic posturography provides a more complete assessment than its static counterpart. There is a larger array of stimuli and peripheral and central sensorial organs are more intensely required. According to Furman et al.^21^, this is a test which can generate information not checked by electronystagmography and rotational tests for the vestibular dysfunction. The tridimensional electromagnetic system in the present study was utilized only in the static assessment; nevertheless, it has a future potential for the employment of more complex protocols, even associated with the force platform.

The Balance Rehabilitation Unit (BRU™), besides utilizing the force platform technology, associated virtual reality in the diagnosis and rehabilitation of patients with balance disorders^22^. This is equipment with good dynamic assessment resources, without anticipations of a similar development for the Polhemus Patriot^®^. Its cost of purchase is still considered high. In a study led by Kasse et al.^23^ with the BRU™ in elderly patients after the repositioning maneuver to treat BPPV, the static monitoring with eyes closed in an unstable surface was more sensitive than those in stable surface with the eyes closed or opened.

In individuals with posterior canal BPPV, the validity of posturography is controversial. According to Çelebisoy et al.^24^, there is a significant increase in sway velocity with eyes closed on unstable surface, and there was a certain correlation with the current paper, which detected considerable changes in the situations of eyes open and closed on an unstable surface. According to Di Girolamo^25^, There are also significant differences in some body balance analysis positions. Authors, such as Chang et al.^26^, did not find important differences when compared to the control group.

Giacomini et al.^27^ noticed that, after the repositioning maneuvers in BPPV patients, there was a persistence of anteroposterior sway, despite the medio-lateral improvement. This is a topic which can be assessed by the Polhemus directly monitoring the mass center and no longer the pressure center.

Upon analyzing the maximum shift of group 1 case volunteers in comparison to the control group, we noticed a statistical difference only in the medio-lateral direction and in the OESS situation (*p* < 0.05), despite the tendency of increase in this variable in all sensorial conditions an in the anteroposterior and mediolateral situations. Other studies carried out with the Polhemus^®^ found a contrary trend: a reduction in body sway amplitude^13^, ^18^; nevertheless, in a population of elderly and obese persons without other associated pathologies. The current sample was made up of volunteers with acute symptoms and with more intense balance disorders, which could justify this finding.

The present study, as well as others which used the same equipment, developed in a safe, fast and reliable way^1^, ^18^, consolidated the concept of future Polhemus Patriot^®^ applications in otorhinolaryngology, in the office, in emergency and even in infirmaries^13^. This new resource may be useful both in initial diagnosis as in the clinical follow up of patients^28^. Revision studies state that the summation of posturography and electronystagmography information associated with that of validated questionnaires increase diagnosis sensitivity^29^.

## CONCLUSION

In the current study, the volunteers in the group of peripheral vestibular dysfunctions detected by electronystagmography and calorimetric tests had a higher body sway in relation to those with positive Epley and Dix-Hallpike maneuvers and a group of healthy volunteers. The system of tridimensional electromagnetic sensors used to assess the balance proved sensitive to detect the differences in body sway between the groups in different sensorial situations, becoming one more tool used to enhance the diagnosis of patients with balance disorders.
